# Suramin Inhibits SARS-CoV-2 Infection in Cell Culture by Interfering with Early Steps of the Replication Cycle

**DOI:** 10.1128/AAC.00900-20

**Published:** 2020-07-22

**Authors:** Clarisse Salgado-Benvindo, Melissa Thaler, Ali Tas, Natacha S. Ogando, Peter J. Bredenbeek, Dennis K. Ninaber, Ying Wang, Pieter S. Hiemstra, Eric J. Snijder, Martijn J. van Hemert

**Affiliations:** a Department of Medical Microbiology, Leiden University Medical Center, Leiden, The Netherlands; b Department of Pulmonology, Leiden University Medical Center, Leiden, The Netherlands

**Keywords:** COVID-19, SARS-CoV-2, suramin, antiviral agents, coronavirus, drug repurposing

## Abstract

The severe acute respiratory syndrome coronavirus 2 (SARS-CoV-2) pandemic that originated in Wuhan, China, in December 2019 has impacted public health, society, the global economy, and the daily lives of billions of people in an unprecedented manner. There are currently no specific registered antiviral drugs to treat or prevent SARS-CoV-2 infections. Therefore, drug repurposing would be the fastest route to provide at least a temporary solution while better, more specific drugs are being developed. Here, we demonstrate that the antiparasitic drug suramin inhibits SARS-CoV-2 replication, protecting Vero E6 cells with a 50% effective concentration (EC_50_) of ∼20 μM, which is well below the maximum attainable level in human serum. Suramin also decreased the viral load by 2 to 3 logs when Vero E6 cells or cells of a human lung epithelial cell line (Calu-3 2B4 [referred to here as “Calu-3”]) were treated. Time-of-addition and plaque reduction assays performed on Vero E6 cells showed that suramin acts on early steps of the replication cycle, possibly preventing binding or entry of the virus. In a primary human airway epithelial cell culture model, suramin also inhibited the progression of infection. The results of our preclinical study warrant further investigation and suggest that it is worth evaluating whether suramin provides any benefit for COVID-19 patients, which obviously requires safety studies and well-designed, properly controlled randomized clinical trials.

## Abstract (precis)

The severe acute respiratory syndrome coronavirus 2 (SARS-CoV-2) pandemic that originated in Wuhan, China, in December 2019 has impacted public health, society, the global economy, and the daily lives of billions of people in an unprecedented manner. There are currently no specific registered antiviral drugs to treat or prevent SARS-CoV-2 infections. Therefore, drug repurposing would be the fastest route to provide at least a temporary solution while better, more specific drugs are being developed.

## INTRODUCTION

In December 2019, local health authorities reported an increasing number of pneumonia cases, rapidly spreading across the city of Wuhan, Hubei province, in China (1). Further analysis showed that the causative agent of this disease was severe acute respiratory syndrome coronavirus 2 (SARS-CoV-2), which is a member of the betacoronavirus genus within the coronavirus family and shares roughly 80% of genetic identity with SARS-CoV (2, 3). Since then, SARS-CoV-2 has spread to 113 countries, leading to a coronavirus pandemic of unprecedented magnitude, with more than 3.5 million confirmed cases globally and more than 240,000 casualties reported by WHO on 5 May 2020 (4).

Coronaviruses are enveloped viruses that possess extraordinarily large (26-to-32-kb) positive-strand RNA genomes (5). SARS-CoV-2 infection often causes only mild disease but can also lead to clinical manifestations such as high fever, cough, dyspnea, myalgia, and headache. Although the majority of cases may be asymptomatic or present mild symptoms with good recovery, some patients experience more-severe outcomes, such as severe pneumonia, respiratory failure, multiple-organ failure, or death (6).

Due to the urgency of the situation, the lack of approved specific antiviral therapy against coronaviruses, and the time it takes to develop the latter through regular preclinical and clinical research, there is great interest in repurposing already approved drugs. This would be a fast track to apply (candidate) therapeutic agents as antivirals to combat SARS-CoV-2 infection, which can be used to fight the virus while better and more specific antivirals are being developed.

Drugs such as ribavirin, remdesivir, favipiravir, and the antimalarial therapeutic chloroquine showed promise in cell culture, and some also appeared to show (modest) effects in early trials in humans, which were not always conducted with the most optimal design (7). However, except for remdesivir (8), more-recent (and more appropriately conducted) clinical trials have suggested that none of these drugs provide substantial benefit in patients and that they should be used with caution due to their potential side effects. Therefore, it appears that options to inhibit SARS-CoV-2 infection are limited and that mainly supportive care and treatments that target the immune system and inflammatory responses can be provided to patients. This stresses the urgency of evaluating additional approved drugs as candidates for use as antiviral therapy against this pathogen.

We now provide evidence showing that suramin can be considered a drug candidate that deserves further assessment, as we found the compound to exhibit antiviral activity against SARS-CoV-2 in relevant cell culture models at concentrations that can be easily reached in human serum (9). Suramin is an antiparasitic drug that is used to treat sleeping sickness caused by trypanosomes. It is a symmetrical polysulfonated compound that was synthesized for the first time around 1916 (10). More recently, we and many others have shown that suramin also has broad-spectrum antiviral effects, as it inhibits HIV (11), hepatitis C virus (12), herpes simplex type-1 virus (13), Zika virus (14), dengue virus (15), chikungunya virus (16), and others.

In the present study, we showed that suramin also exhibits antiviral activity against SARS-CoV-2 in cell culture, inhibiting an early step in the replication cycle, i.e., likely binding and/or entry. The compound had an 50% effective concentration (EC_50_) of 20 μM in Vero E6 cells and showed a more than 2 log viral load reduction when infected human Calu-3 2B4 airway epithelial (AE) cells (referred to here as “Calu-3” cells) were treated. Finally, suramin reduced SARS-CoV-2 progression of infection in well-differentiated primary human airway epithelial (HAE) cells cultured at the physiological air-liquid interface (ALI). It is important to stress that suramin can have serious side effects and that our results should not be directly translated as representative of efficacy against SARS-CoV-2 in humans and do not yet guarantee a benefit to the patient. However, our results make suramin an interesting candidate for further evaluation in in-depth preclinical studies (e.g., investigations into formulation, mode of administration, and pharmacokinetics and in other *ex vivo* models) and suggest that suramin could ultimately be explored in carefully performed and properly controlled clinical trials for the treatment of COVID-19 patients.

## RESULTS

### Suramin inhibits SARS-CoV-2 replication in Vero E6 cells.

**FIG 1:**
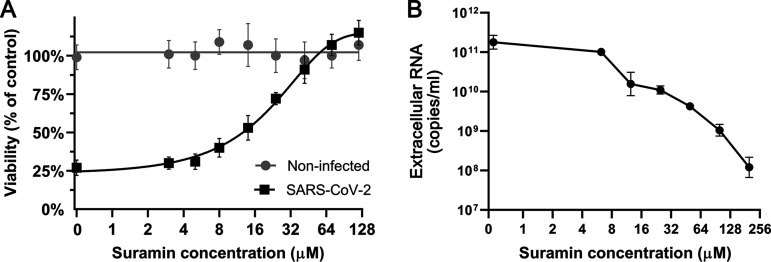
Suramin inhibits SARS-CoV-2 replication in Vero E6 cells. (A) CPE reduction assay. Vero E6 cells were treated with 1.7-fold serial dilutions of suramin and subsequently infected with SARS-CoV-2 at an MOI of 0.015. After further incubation in medium with compound, cell viability was measured by MTS assay at 3 days postinfection. The viability of noninfected suramin-treated cells was measured in parallel to assess toxicity (3 independent experiments performed in quadruplicate). Mean values ± standard deviations (SD) are shown. The nonlinear regression curves resulting from curve fitting are depicted as solid lines. (B) Viral load reduction assay. Vero E6 cells were treated with different concentrations of suramin, followed by infection at an MOI of 1 and further incubation in medium with compound. After 16 h, supernatants were harvested and the viral load was determined by quantification of extracellular SARS-CoV-2 RNA by an internally controlled multiplex RT-qPCR targeting the RdRp coding region (*n* = 3). Mean values ± SD are shown.

To determine if suramin could protect cells from SARS-CoV-2 infection and to evaluate its toxicity, a cytopathic effect (CPE) reduction assay was performed. Vero E6 cells were pretreated with serial dilutions of suramin, were infected with SARS-CoV-2, and were kept in the medium with compound for 72 h. Suramin protected infected cells from SARS-CoV-2-induced cell death in a dose-dependent manner, with an EC_50_ of 20 ± 2.7 μM (Fig. 1A). In parallel, noninfected cells were treated with the same concentrations of suramin in order to assess the compound’s toxicity. No toxicity was observed over the range of concentrations that was used in these antiviral assays. Cell viability dropped to 67% only at 5 mM, resulting in a 50% cytotoxic concentration (CC_50_) value of >5 mM (16). Therefore, suramin inhibits SARS-CoV-2 with a selectivity index (SI) higher than 250.

To more directly measure the inhibition of viral replication by suramin, viral load reduction assays were performed. Vero E6 cells were pretreated with increasing concentrations of suramin and infected with SARS-CoV-2 at a multiplicity of infection (MOI) of 1, followed by further incubation in medium with compound. At 16 h postinfection (hpi), supernatant was harvested to determine the viral load by quantifying the levels of extracellular viral RNA by real-time quantitative PCR (RT-qPCR) targeting the RNA-dependent RNA polymerase (RdRp) coding region (Fig. 1B). The supernatant of untreated infected cells contained 10^11^ copies/ml of viral RNA. RT-qPCR revealed that the RNA levels decreased upon suramin treatment in a dose-dependent manner, showing a 3-log reduction at the highest concentration tested (200 μM) (Fig. 1B). Together, these results indicated that suramin protects Vero E6 cells from the SARS-CoV-2-induced cytopathic effect and that it reduces the viral load in these cells.

### Suramin reduces the viral RNA load and infectious virus yield in cultured human lung epithelial cells.

**FIG 2:**
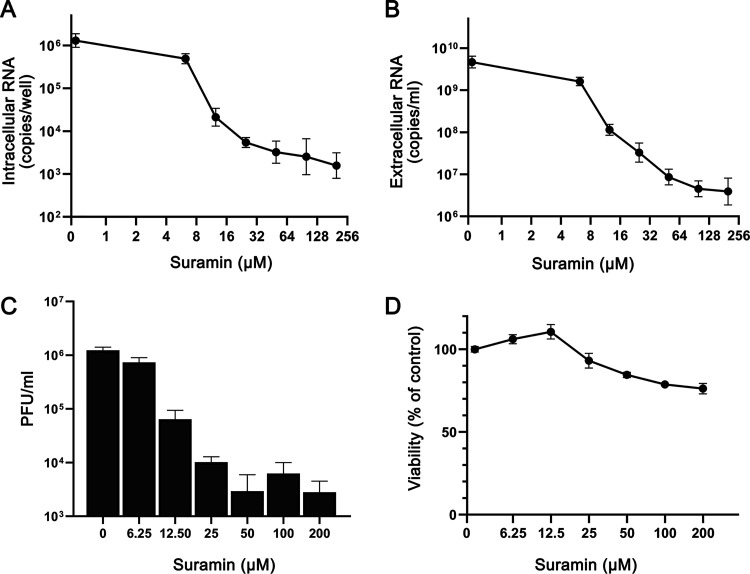
Suramin decreases levels of intra- and extracellular viral RNA and infectious progeny in infected Calu-3 cells. Calu-3 cells were infected with SARS-CoV-2 in the presence of suramin, followed by washing and continued treatment with 0 to 200 μM suramin. (A) Intracellular viral RNA copy numbers at 21 hpi, determined by internally controlled multiplex RT-qPCR targeting the SARS-CoV-2 RdRp coding region and using the housekeeping gene PGK1 for normalization. (B) Extracellular viral RNA levels at 21 hpi, quantified by RT-qPCR. (C) Viral load in the supernatant at 21 hpi as determined by plaque assay on Vero E6 cells. (D) Viability of uninfected Calu-3 cells treated with various concentrations of suramin measured by MTS assay in parallel to the infection (*n* = 3). Mean values ± SD are shown.

To assess the antiviral effect of suramin in a more relevant model, human lung epithelial cells (Calu-3) were infected with 2 × 10^4^ PFU of SARS-CoV-2 in the presence of 0 to 200 μM suramin for 1 h. After removal of the inoculum and washing of the cells, incubation was continued in medium with suramin (0 to 200 μM) for 20 h. At 21 hpi, RNA was isolated from cells and supernatant and the viral titer in the supernatant was determined by plaque assay. We observed a strong dose-dependent reduction in intracellular (Fig. 2A) and extracellular (Fig. 2B) viral RNA levels in suramin-treated samples. The extracellular viral RNA levels showed a 3-log reduction at 200 μM, while intracellular viral RNA levels decreased by 2-log. Panels A and B of Fig. 2 show the results of RT-qPCRs targeting the RdRp coding region, but similar reductions in copy numbers were observed with RT-qPCRs targeting the SARS-CoV-2 N protein gene (an assay that also detects all subgenomic RNAs), although in that case absolute copy numbers—as expected—were higher than for genomic RNA (data not shown). Plaque assays confirmed that treatment with 200 μM suramin led to an almost 3-log drop in infectious progeny titers from infected-Calu-3 cells (Fig. 2C).

Cytotoxicity assays performed in parallel in noninfected Calu-3 cells showed that suramin was slightly more toxic to these cells than to Vero E6 cells, although cell viability remained above 80% even at the highest dose tested (Fig. 2D). Higher concentrations of suramin were tested on Calu-3 cells (data not shown), but viability was not reduced to levels under 50%, leading us to conclude that the CC_50_ is >500 μM. The suramin EC_90_ (i.e., the suramin concentration that reduced the extracellular genome copy numbers by 90%) was 9 μM. Using these values, we calculated a therapeutic index (CC_50_ divided by EC_90_) value of >55. Together, these results suggest that suramin is a potent SARS-CoV-2 inhibitor with high selectivity also in human lung cells.

### Suramin acts at the early steps of viral replication.

**FIG 3:**
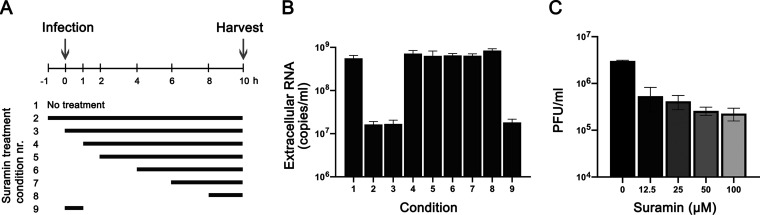
Suramin inhibits the early steps of SARS-CoV-2 replication. (A) Schematic representation of the time-of-addition experiment and the different treatment intervals. (B) At 10 hpi, supernatant was harvested and extracellular viral RNA levels were determined by RT-qPCR targeting the RdRp coding region (*n* = 3). (C) Vero E6 cells were infected with SARS-CoV-2 in the presence of various concentrations of suramin. Suramin was present only during the 1 h of infection, and after 1 h, cells were incubated in overlay medium without suramin. After 3 days, cells were fixed and stained and plaques were counted (*n* = 3). Mean values ± SD are shown.

To determine which step of viral replication is affected by suramin, we performed a time-of-addition assay. Cells were infected with SARS-CoV-2 (MOI of 1) and treated with 100 μM suramin over different time intervals, as schematically depicted in Fig. 3A. Treatment was initiated 1 h before infection or at 0, 1, 2, 4, 6, or 8 hpi, and suramin remained present until 10 hpi, when supernatants were harvested to determine viral load by RT-qPCR targeting the RdRp coding region. In one sample, suramin was present for only 60 min during the time of infection. After 1 h, virus inoculum was removed and cells were washed three times with phosphate-buffered saline (PBS), followed by incubation in medium with or without suramin. At 10 hpi, supernatant was collected to evaluate the levels of viral RNA (Fig. 3B). When suramin treatment was initiated 1 h before infection (−1 h) or at the time of infection (0 h) a 2-log reduction in viral RNA levels was observed. Treatments that started later than 1 hpi did not inhibit viral replication, as viral RNA levels similar to those seen with the nontreated control were observed. Treatment only during the infection (0 to 1 h) resulted in the same 2-log reduction in viral RNA load as that seen with the 0-to-10-h treatment, indicating that suramin inhibits an early step of the replication cycle, likely viral entry.

To confirm suramin’s early inhibitory effect, we performed a plaque reduction assay by infecting Vero E6 cells with serial dilutions of SARS-CoV-2 in the presence of increasing concentrations of suramin, which was present only during the 1 h of infection. After infection, cells were washed 3 times with PBS and were incubated with overlay medium without suramin. After 3 days, cells were fixed and stained and plaques were counted. Suramin caused a dose-dependent reduction in the number of plaques, and even at the lowest suramin concentration (12.5 μM) titers were already reduced by almost 1 log (Fig. 3C). These results suggest that suramin inhibits SARS-CoV-2 infection by interfering with an early step of the replication cycle.

### Suramin inhibits SARS-CoV-2 replication in a primary human epithelial airway cell infection model.

**FIG 4:**
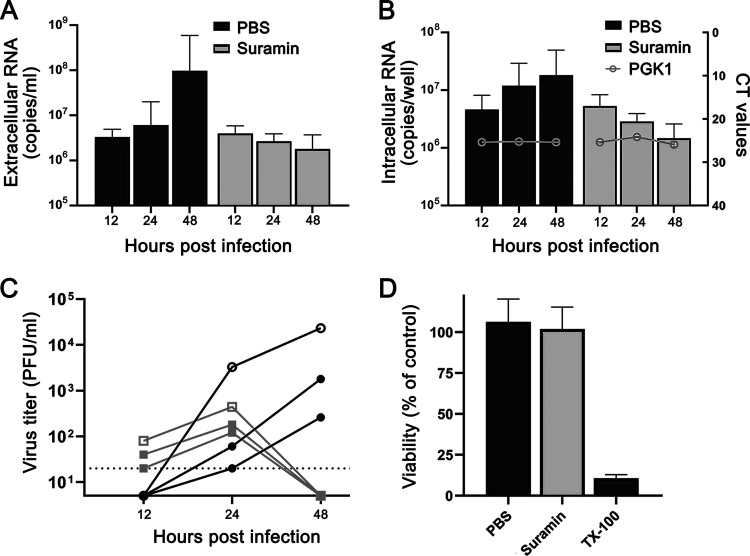
Suramin inhibits progression of SARS-CoV-2 infection in primary human airway epithelial cells. HAE cells were infected with 30,000 PFU of SARS-CoV-2 (estimated MOI of 0.1), and they were treated with 50 μl PBS or 50 μl of 100 μM suramin at 12 hpi and 24 hpi. (A) Levels of extracellular viral RNA were determined by RT-qPCR at 12, 24, and 48 hpi (*n* = 3). (B) Intracellular viral RNA levels were determined with an internally controlled multiplex RT-qPCR (bars, left axis). Levels of the housekeeping gene PGK1 were analyzed for normalization and to check for signs of cell death (gray lines, right axis). (C) Infectious virus titers in apical wash samples collected at 12, 24, and 48 hpi, determined by plaque assay. Open symbols represent mixed-donor inserts, and closed symbols represent single-donor inserts. Black symbols represent PBS-treated samples, and gray symbols represent suramin-treated samples. The dotted line at 20 PFU/ml indicates the limit of detection. (D) Viability of suramin-treated cells evaluated by MTS assay, using treatment with 0.1% Triton X-100 (TX-100) as a positive control for cell toxicity (*n* = 6). Mean values ± SD are shown.

Primary HAE cell cultures mimic the morphological and physiological features of the human conducting airway, arguably representing the most relevant *ex vivo* model for human coronavirus research (17–19). For that reason, we decided to also evaluate the antiviral effect of suramin in this model. HAE cells were differentiated by culture at the air-liquid interface to achieve mucociliary differentiation. HAE cultures were infected for 1 h with 30,000 PFU of SARS-CoV-2 (estimated MOI of 0.1 based on the number of cells present on an insert), followed by washing with PBS. At 12 and 24 hpi, the cultures were treated on the apical side with either 50 μl of 100 μM suramin or 50 μl PBS. The HAE cell culture apical side was washed with PBS for 10 min at 37°C, and this supernatant was harvested at 12, 24, and 48 hpi to analyze the viral load by RT-qPCR targeting the RdRp coding region. RNA was also isolated from cells to quantify the levels of intracellular viral RNA and the housekeeping gene PGK1. RT-qPCR analysis of extracellular viral RNA levels showed that approximately 10^7^ copies/ml of viral RNA remained at 1 hpi. The viral load in the supernatant was not significantly increased at 12 and 24 hpi in untreated cells, while a more than 1 log increase in viral RNA copies was observed at 48 hpi. This is indicative of (very modest) viral replication in PBS-treated cells. The cultures that were treated with suramin displayed no increase in viral load in the supernatant but instead even showed a slight decrease in copy numbers, suggesting that viral replication had not progressed in the treated cells. At 48 hpi, the supernatant of suramin-treated cells showed 2-log-lower SARS-CoV-2 genome copy numbers than PBS-treated control cells (Fig. 4A). The levels of intracellular viral RNA displayed the same trend, with a decrease in viral RNA in suramin-treated samples compared to an increase in viral RNA in PBS-treated samples (Fig. 4B). A 1-log difference, from 10^7^ to 10^6^ copies per transwell, was observed at 48 hpi between suramin- and PBS-treated cells (Fig. 4B). The levels of the PGK1 housekeeping gene remained stable in all samples, suggesting that the reduction in the number of viral RNA copies was not due to cell death. Moreover, cell viability measured by 3-(4,5-dimethylthiazol-2-yl)-5-(3-carboxymethoxyphenyl)-2-(4-sulfophenyl)-2H-tetrazolium (MTS) assay (Fig. 4D) and lactate dehydrogenase (LDH) assay (data not shown) suggested that the suramin treatment (compared to PBS treatment) had no measurable cytotoxic effect on HAE cells. To determine the effect of suramin on infectious progeny released by HAE cells, we performed a plaque assay with the harvested supernatant. The infectious virus yield of inserts containing cells from single donors was lower than those of transwells containing a mix of different donors (Fig. 4C). This difference might have been due to donor-specific (genetic) differences and/or might have been a consequence of the fact that 4 × 10^4^ cells were seeded on single-donor transwells whereas 15 × 10^4^ cells were seeded on donor mix transwells. At 24 hpi, a modest difference was observed between the levels of infectious progeny released by cells treated with PBS (3.3 × 10^3^ PFU/ml) and suramin-treated cells (4.4 × 10^2^ PFU/ml) on mixed-donor inserts (Fig. 4C). However, at 48 hpi, the supernatant of PBS-treated cells contained over 10^4^ PFU/ml (for mixed donors), while no infectious virus was found in any of the suramin-treated samples (limit of detection, 20 PFU/ml). This suggests that suramin reduces the progression of infection in a HAE cell culture infection model.

## DISCUSSION

The emergence of SARS-CoV-2 and its enormous impact on public health, society, the global economy, and the lives of billions around the globe have prompted a multitude of efforts to develop vaccines and antivirals. Due to the lengthy process of development of new and specific antivirals, there is a particular interest in repurposing existing drugs for treatment of the COVID-19 disease. This could provide a temporary solution while better and more-specific drugs are being developed. Several small-molecule compounds such as chloroquine, hydroxychloroquine, favipiravir, and remdesivir have shown some efficacy against SARS-CoV-2 *in vitro* (7). However, despite promising results in preclinical studies, recent clinical trials (20, 21) suggested that these compounds, with the exception of remdesivir (8), do not provide much benefit to COVID-19 patients and could actually be dangerous due to possible side effects. This leaves us currently empty-handed and in search of other approved drugs that might be repurposed. As an already approved antiparasitic drug, suramin would be one of the candidates for fast development of a treatment for COVID-19. Antiviral activity of suramin against RNA viruses was reported earlier by us and several other groups (16, 22–24), and the compound was and is also being evaluated in several clinical trials for other diseases, providing some evidence for its safety for therapeutic use. However, suramin can also cause several side effects, including those which caused previous HIV trials with seriously ill patients to halt (25); therefore, caution is advised and it is crucial to conduct well-controlled randomized trials before any conclusions on possible benefits for COVID-19 patients can be drawn. Thus far, no studies have reported a potential antiviral effect of suramin against coronaviruses.

In this study, we assessed the antiviral activity of suramin against the newly emerged virus strain SARS-CoV-2. Suramin offered full protection against SARS-CoV-2-induced cell death in Vero E6 cells and inhibited the virus with an EC_50_ of 20 μM and a SI of >250 (Fig. 1). Suramin treatment of infected Vero E6 cells led to a reduction in extracellular viral RNA levels of up to 3 log. The highest concentration of compound that was used proved harmless to the cells; also, cytotoxicity was observed previously only above 5 mM (16). Suramin also displayed antiviral efficacy in a human lung epithelial cell line, and we observed a >2 log reduction in levels of infectious virus progeny in suramin-treated cells (CC_50_/EC_90_ = >55).

Suramin was previously described as having the potential to inhibit several stages of virus replication by acting on different targets (16, 26). To assess which step in the SARS-CoV-2 replication cycle is affected by suramin treatment, we performed a time-of-addition assay on Vero E6 cells. We observed that pretreatment with suramin as well as addition during the first hour of infection resulted in a marked decrease in the levels of viral RNA in the supernatant, while treatments initiated after the first hour of infection showed no significant effect on virus replication, suggesting that suramin acts on an early replication step, possibly binding or entry. In addition, SARS-CoV-2 infectivity was decreased in plaque assays, when suramin was present only in the inoculum during infection, concordant with an effect on the early stages of infection (Fig. 3). This is in agreement with other studies that also reported on the inhibition of virus binding or entry by suramin (16, 23, 27). Several studies have suggested that negatively charged suramin molecules can bind to positive charges on virions (22, 28, 29). Our data suggest that the antiviral effect of suramin is primarily due to inhibition of an early step in the SARS-CoV-2 replication cycle, and we suspect that this is due to binding of the compound to SARS-CoV-2 virions, interfering with their binding to the cell receptor and possibly also inhibiting fusion.

Finally, we evaluated the effect of suramin in a more relevant model of differentiated primary human airway epithelial (HAE) cells cultured and infected at the physiologically relevant air-liquid interface. We infected these cells with a relatively low dose of virus (estimated MOI of 0.1) and treated them with suramin by applying a 50-μl volume of 100 μM suramin on the apical side at 12 and 24 hpi. This would allow us to follow spread of the viral infection and assess whether suramin is able to block progression of infection in this “treatment model.” HAE cell cultures represent a composition of highly differentiated cells mainly containing basal, goblet, club, and ciliated cells and hence represent an air-liquid interface that mimics the lung airway epithelium (30, 31). In a recent study, it was shown that SARS-CoV-2, like SARS-CoV, uses human angiotensin-converting enzyme 2 (ACE2) receptors for attachment in these human airway cells. Blocking of the host protease TMPRSS2, which is important for priming the fusion activity of the spike protein, also inhibited infection in lung cells (32). To address the variation of these proteins and the diversity of primary human airway cells within patients, we made use of HAE cultures that were obtained from different donors. Notably, we observed differences in the levels of susceptibility of cultures from different donors, among which HAE cultures from mixed donors showed higher titers. A total of 4 × 10^4^ cells were seeded for single-donor HAE cultures, while 15 × 10^4^ cells were seeded for mixed-donor HAE cultures, which could be one explanation for the observed differences. Moreover, different donors might have differing levels of susceptibility to infection, possibly caused by genetic differences and differences in cell differentiation and composition (33).

Administration of 100 μM suramin on the apical side of the HAE cells did not appear to cause cytotoxic effects in our study (Fig. 4). In our HAE model for progression of SARS-CoV-2 infection, we infected cells at a low MOI and observed a modest (∼200-fold) increase in viral load by 48 hpi in PBS-treated cultures compared to the levels observed at 12 hpi. Although the increase in viral load was rather modest in control cells, we found no evidence for progression of the infection in suramin-treated cultures, as indicated by the SARS-CoV-2 RNA levels, which remained equal to those seen at 1 hpi or even decreased over time. Moreover, the infectious-progeny titer increased over time in PBS-treated HAE cultures and had reached over 10^4^ PFU/ml by 48 hpi, while in suramin-treated HAE cells, infectious progeny showed a modest increase at 24 hpi (10-fold lower than that seen with the PBS-treated cells) and dropped to undetectable levels at 48 hpi. Since suramin-containing samples needed to be diluted 20-fold to exclude interference with the plaque assay, the limit of detection would be 20 PFU/ml. Even with this limit of detection, the supernatant collected from suramin-treated HAE cells contained at least 1,000 times less virus than that from PBS-treated cells. Much higher titers were obtained with HAE cultures from mixed donors than with those from single donors, but the inhibitory effect of suramin was also observed with single-donor cultures. Overall, despite the modest level of infection in control cells, our results suggest that, also in the HAE infection model, suramin had an inhibitory effect on the progression of the SARS-CoV-2 infection.

Our study demonstrated that suramin inhibited SARS-CoV-2 replication in various cell culture models and at clinically achievable concentrations (after intravenous [i.v.] administration, serum levels of >10× the EC_50_ were achieved). Due to its mode of action, treatment of patients with suramin might require administration at an early stage, although it might also prevent spread of the virus in the lungs of already symptomatic patients or might prevent spread from respiratory tract to other organs. It might even be possible to use suramin to prevent virus spreading in the nasopharynx, which appears to be the first site of infection (34–36). Standard treatment with suramin is done by intravenous administration, which would also be an option for seriously ill COVID-19 patients who are in intensive care but is not ideal for other patients. As a negatively charged compound, suramin binds to various proteins and is poorly taken up by diffusion across the cell membrane, although it can be taken up by endocytosis (26). This poor uptake of suramin into cells might not necessarily be a problem for efficacy against SARS-CoV-2, as it is expected to block the virus systemically and in the extracellular environment. Hypothetically, suramin administration into the respiratory tract in an aerosolized form could be even considered, although that would require new safety studies.

In conclusion, our preclinical study showed that suramin inhibited SARS-CoV-2 replication in cell culture, likely by preventing entry. Suramin also appeared to prevent progression of SARS-CoV-2 infection in a human airway epithelial cell culture model. This is only the first step toward evaluating whether suramin treatment could provide any benefit to COVID-19 patients. Further studies should carefully evaluate different formulations, routes of administration, pharmacokinetics, and possible adverse effects in cell culture and *ex vivo* models. Suramin could have serious side effects in patients. Ultimately, the clinical benefits of suramin for the treatment of COVID-19 patients should be evaluated in carefully performed and properly controlled clinical trials.

## MATERIALS AND METHODS

### Cell lines, virus, and compound.

Vero E6 cells were maintained in Dulbecco’s modified Eagle’s medium (DMEM; Lonza), supplemented with 8% fetal calf serum (FCS; Bodinco), 2 mM l-glutamine, 100 IU/ml of penicillin, and 100 μg/ml of streptomycin (Sigma-Aldrich). The human lung epithelial cell line Calu-3 2B4 (referred to here as Calu-3 cells) was maintained as described previously (37). Primary human airway epithelial (HAE) cell cultures were established at the Department of Pulmonology of the Leiden University Medical Center (LUMC), and their culture and infection are described below. All cell cultures were maintained at 37°C in an atmosphere of 5% CO_2_ and 95% to 99% humidity. Infections were performed in Eagle’s minimal essential medium (EMEM; Lonza) with 25 mM HEPES (Lonza), further supplemented with 2% FCS, l-glutamine (Sigma-Aldrich), and antibiotics.

The clinical isolate SARS-CoV-2/Leiden-0002 was isolated from a nasopharyngeal sample at LUMC, and its sequence and characterization will be described elsewhere (unpublished data; GenBank accession nr. MT510999). SARS-CoV-2/Leiden-0002 was passaged twice in Vero E6 cells, and virus titers were determined by plaque assay as described before (38). Working stocks yielded titers of 5 × 10^6^ PFU/ml. All experiments with infectious SARS-CoV-2 were performed in a biosafety level 3 facility at the LUMC.

Suramin was purchased from Sigma-Aldrich and was dissolved in Milli-Q water and stored at −20°C. Addition of compound to Vero E6 and Calu-3 cells was done in infection medium and in PBS for HAE cultures.

### Human airway epithelial cell (HAE) cultures.

HAE cells were cultured as previously described (39). Briefly, primary bronchial epithelial cells (PBEC) were isolated from tumor-free resected human bronchial tissue from patients undergoing resection surgery for lung cancer at the LUMC. Use of such lung tissue that became available for research within the framework of patient care was in line with the “Human Tissue and Medical Research Code of Conduct for Responsible Use” (2011) (www.federa.org), which describes the opt-out system for coded anonymous further use of such tissue. To achieve mucociliary differentiation, primary bronchial epithelial cells (PBEC) were cultured at the air-liquid interface (ALI) for 21 days as previously described (39, 40). In brief, expanded HAE cells (5 × 10^4^ cells per donor) from 3 donors at passage 2 were combined and were seeded on 12-well transwell membranes (Corning Costar), which were coated with a mixture of bovine serum albumin (BSA), collagen type 1, and fibronectin. In addition, 4 × 10^4^ cells from two individual donors were seeded on separate sets of transwell membranes. BEpiCM-b:DMEM (B/D)-medium (1:1) (supplemented with 12.5 mM HEPES, bronchial epithelial cell growth supplement, antibiotics, 1 nM EC23 [retinoic acid receptor agonist], and 2 mM GlutaMAX) was used as described previously (40). After confluence was reached, cells were cultured at the ALI in complete medium with 50 nM EC23 for 21 days. The mucociliary differentiated cultures were characterized by detection of high transepithelial electrical resistance (TEER; >500 Ω·cm^2^), visible cilium beating and mucus production. Before infection, cells were incubated overnight in a BEpiCM-b/DMEM 1:1 medium mixture from which epidermal growth factor (EGF), bovine pituitary extract (BPE), BSA, and hydrocortisone were omitted but that did contain antibiotics (starvation medium).

### RNA isolation and quantitative RT-PCR (RT-qPCR).

RNA was isolated from cell culture supernatants and cell lysates using TriPure isolation reagent (Sigma-Aldrich). Equine arteritis virus (EAV) in AVL lysis buffer (Qiagen) was spiked into the reagent as an internal control for extracellular RNA samples. The cellular household gene PGK1 served as a control for intracellular RNA. Primers and probes for EAV and PGK1 and the normalization procedure were described before (38). Viral RNA was quantified by RT-qPCR using TaqMan Fast Virus 1-step master mix (Thermo Fisher Scientific). Primers and probes were used as described previously (41) but with modifications that resulted in the following primer and probe sequences: for SARS-CoV-2 N-Gene, Fwd CACATTGGCACCCGCAATC, Rev GAGGAACGAGAAGAGGCTTG, and probe YakYel (5′ Yakima Yellow)-ACTTCCTCAAGGAACAACATTGCCA-black hole quencher 1 (BHQ1); for RdRp-Gene, Fwd GTGARATGGTCATGTGTGGCGG, Rev CARATGTTAAASACACTATTAGCATA, and probe FAM (6-carboxyfluorescein)-CCAGGTGGAACMTCATCMGGWGATGC-BHQ1. A standard curve of 10-fold serial dilutions of a T7 RNA polymerase-generated *in vitro* transcript containing the RT-qPCR target sequences was used for absolute quantification. A RT-qPCR program of 5 min at 50°C and 20 s at 95°C, followed by 45 cycles of 5 s at 95°C and 30 s at 60°C, was performed on a CFX384 Touch real-time PCR detection system (Bio-Rad).

### Cytopathic effect (CPE) reduction assay.

CPE reduction assays were performed as described previously (42). Briefly, Vero E6 cells were seeded in 96-well cell culture plates at a density of 10^4^ cells per well. Cells were incubated with 1.7-fold serial dilutions of suramin starting from a concentration of 120 μM for 30 min. Subsequently, cells either were mock infected (for analysis of cytotoxicity of the compound) or were infected with 300 PFU of virus per well (MOI of 0.015) in a total volume of 150 μl of medium with compound. Cell viability was assessed 3 days postinfection by MTS assay using a CellTiter 96 aqueous nonradioactive cell proliferation kit (Promega), and absorption was measured at 495 nm with an EnVision multilabel plate reader (PerkinElmer). The 50% effective concentration (EC_50_ [the concentration required to inhibit virus-induced cell death by 50%]) and the 50% cytotoxic concentration (CC_50_) (the concentration that reduces the viability of uninfected cells to 50% of that of untreated control cells) were determined using nonlinear regression with GraphPad Prism v8.0.

### Viral load reduction assays.

Cells were seeded in 96-well cell culture plates at a density of 10^4^ (Vero E6) or 6 × 10^4^ (Calu-3) cells per well in 100 μl culture medium. As a control to determine the amount of residual virus after removal of the inoculum and washing, cells in some wells were killed with 70% ethanol (followed by washing with PBS). Vero E6 and Calu-3 cells were preincubated for 30 min with 2-fold serial dilutions of a starting concentration of 200 μM suramin and subsequently infected with 2 × 10^4^ PFU of SARS-CoV-2 (MOI of 1 on Vero E6 cells) in 50 μl medium with compound. After 1 h, cells were washed three times with PBS and 100 μl of medium with compound was added. For analysis of viral RNA, supernatant was harvested from Vero E6 cells at 16 hpi and from Calu-3 cells at 21 hpi. Intracellular RNA was collected by lysing the cells in 150 μl TriPure reagent. Analysis of viral progeny in supernatant from Calu-3 cells was performed by plaque assay on Vero E6 cells (38). The potential cytotoxicity of the compound was tested in parallel on uninfected cells using the MTS assay (Promega) as described for the CPE reduction assay.

### Entry inhibition plaque reduction assay.

One day before infection, Vero E6 cells were seeded in 6-well cell culture plates at a density of 3.5 × 10^5^ cells per well in 2 ml medium. Serial dilutions (10^−2^-fold to 10^−5^-fold) of a SARS-CoV-2 stock were prepared in medium containing 100, 50, 25, 12.5, 6.25, or 0 μM suramin. These were used as inocula to infect the Vero E6 cells in 6-well clusters. After 1 h at 37°C, the inoculum was removed and cells were incubated in Avicel-containing overlay medium without suramin for 3 days, after which they were fixed with 3.7% formaldehyde and stained with crystal violet and plaques were counted (38).

### Time-of-addition assay.

Vero E6 cells were seeded in 24-well clusters at a density of 6 × 10^4^ cells per well. The next day, the cells were treated with 100 μM suramin during the time intervals indicated in Fig. 3 and were infected at an MOI of 1. Supernatant was harvested at 10 hpi for quantification of viral RNA by RT-qPCR.

### Infection and suramin treatment of HAE cells.

The apical sides of HAE cell cultures were washed 3 times with 200 μl PBS for 10 min at 37°C on the day before infection to remove excess mucus. Washing was repeated once before cells were infected on the apical side with 3 × 10^4^ PFU SARS-CoV-2 (estimated MOI of 0.1) mixed in 200 μl of PBS. After incubation at 37°C for 1 h, the inoculum was removed and cells were washed three times with warm PBS. The last wash was analyzed to determine the amount of input virus that remained in the transwell after infection and washing (baseline). The apical side was treated with 100 μM suramin mixed in 50 μl of PBS at 12 and 24 hpi (after first collecting a 200-μl PBS wash volume to determine viral load). Control wells were treated with 50 μl of PBS. The experiment was done in triplicate, with one insert (transwell) containing a mix of cells from 3 donors and two “single-donor” inserts seeded with cells from two different donors. Supernatants were collected from infected PBS-treated cells and infected suramin-treated cells at 12, 24, and 48 hpi by incubating the apical side with 200 μl PBS for 10 min at 37°C and collecting the supernatant. This supernatant was used for quantification of viral RNA by RT-qPCR and of viral load (infectivity) by plaque assay on Vero E6 cells. At each time point, cell lysates were collected from inserts by adding 750 μl TriPure reagent. Assessment of the potential cytotoxicity of the 48-h suramin treatment, compared to PBS treatment, was done with uninfected cells by MTS assay (Promega) and LDH assay (CytoTox 96 nonradioactive cytotoxicity assay; Promega) according to the manufacturer’s instructions.
